# Predicting Colloidal Interaction Parameters from Small-Angle X-ray Scattering Curves Using Artificial Neural Networks and Markov Chain Monte Carlo Sampling

**DOI:** 10.1021/jacsau.4c00368

**Published:** 2024-09-09

**Authors:** Kelvin Wong, Runzhang Qi, Ye Yang, Zhi Luo, Stefan Guldin, Keith T. Butler

**Affiliations:** † Department of Chemical Engineering, University College London, Torrington Place, London WC1E 7JE, U.K.; ‡ Yusuf Hamied Department of Chemistry, Centre for Misfolding Diseases, University of Cambridge, Lensfield Road, Cambridge CB2 1EW, U.K.; § Langmu Bio, Building 2, 112 Jinjiadulu, Yuhang, Hangzhou 311112, China; ∥ Guangdong Provincial Key Laboratory of Advanced Biomaterials, Department of Biomedical Engineering, Southern University of Science and Technology, Shenzhen 518055, China; # Department of Chemistry, University College London, Kathleen Lonsdale Building, Gower Place, London, WC1E 6BS, U.K.; ⬢ Department of Life Science Engineering, Technical University of Munich, Gregor-Mendel-Straße 4, 85354 Freising, Germany; ⬡ TUMCREATE, 1 CREATE Way, #10-02 CREATE Tower, 138602, Singapore

**Keywords:** small-angle scattering, structure factor, colloidal interactions, machine learning, Markov chain Monte Carlo sampling

## Abstract

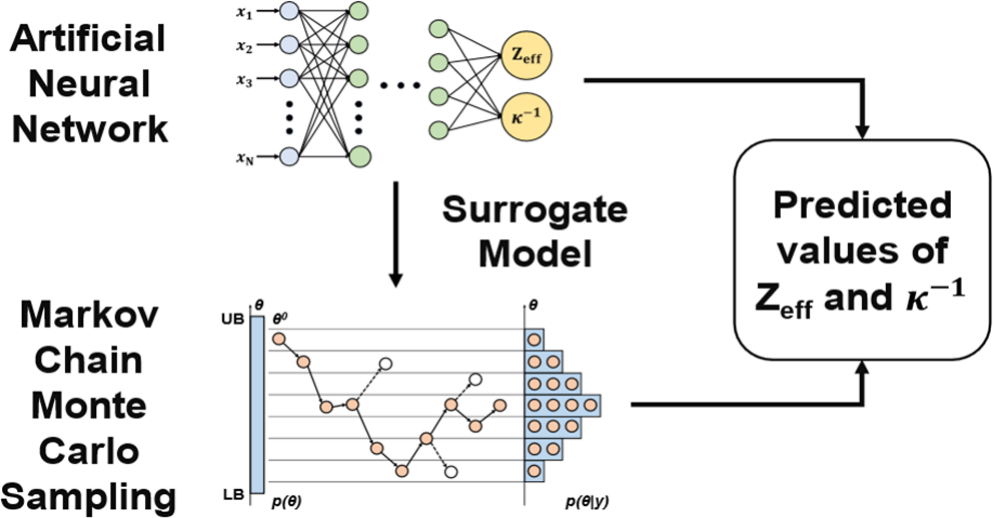

Small-angle X-ray scattering (SAXS) is a characterization technique that allows for the study of colloidal interactions by fitting the structure factor of the SAXS profile with a selected model and closure relation. However, the applicability of this approach is constrained by the limited number of existing models that can be fitted analytically, as well as the narrow operating range for which the models are valid. In this work, we demonstrate a proof of concept for using an artificial neural network (ANN) trained on SAXS curves obtained from Monte Carlo (MC) simulations to predict values of the effective macroion valency (*Z*_eff_) and the Debye length (κ^–1^) for a given SAXS profile. This ANN, which was trained on 200,000 simulated SAXS curves, was able to predict values of *Z*_eff_ and κ^–1^ for a test set containing 25,000 simulated SAXS curves, where most predicted values had errors smaller than 20%. Subsequently, an ANN was used as a surrogate model in a Markov chain Monte Carlo sampling algorithm to obtain maximum a posteriori estimates of *Z*_eff_ and κ^–1^, as well as the associated confidence intervals and correlations between *Z*_eff_ and κ^–1^ for an experimentally obtained SAXS profile.

## Introduction

The Derjaguin–Landau–Verwey–Overbeek (DLVO) theory is a long-standing model in colloid science, which explains colloidal interactions as a sum of attractive van der Waals forces and repulsive electrostatic forces.^1,2^ While the DLVO theory has been widely successful in explaining and predicting colloidal stability for inorganic colloidal particles with simple surface chemistries,^3^ it is becoming increasingly clear that the existing DLVO theory is insufficient for explaining phenomena related to biological colloids, such as protein folding^4^ and the existence of the Hofmeister series.^5^ While researchers across various disciplines have made efforts to extend the DLVO theory by including extended-DLVO (xDLVO) forces such as steric stabilization^6^ and hydration forces,^7^ a unified xDLVO theory capable of fully rationalizing the observed nanoscale phenomenon remains elusive.

Metallic nanoparticles capped with a self-assembled monolayer of organic ligands are a promising model system to study colloidal interactions due to the overall similarity in size and shape between sub-10 nm functionalized metal nanoparticles and globular proteins.^8^ The pronounced scattering length densities of the metal cores in these hybrid inorganic–organic colloids also make them a suitable model system for small-angle X-ray scattering (SAXS) studies.^10^ In addition, these nanoparticles can be engineered to mimic protein surfaces, which are amphiphilic and chemically heterogeneous, by varying the types and mixtures of ligands which form the self-assembled monolayer.^9^ To this end, the gold nanoparticle (AuNP) synthesis protocol by Yang et al. allows for facile tuning of ligand shell composition by varying the ratio of capping ligands.^11^

SAXS is a technique which has been used to study colloidal interactions as information about the spatial configuration of colloidal particles in solution is captured within the structure factor, *S(q)*, of the SAXS scattering profile.^12^ However, the *S(q)* profile is typically largely featureless, which complicates direct parameter extraction from the *S(q)* profile due to the lack of distinct features such as peaks.^13^ Thus, analysis of SAXS curves is typically carried out by fitting the obtained curves using either the Ornstein–Zernike (OZ) integral equation theory or Monte Carlo (MC) simulations. The OZ theory is a thermodynamically consistent theory that can provide an analytical solution for a chosen model given an appropriately selected closure relation with a minimal computational cost. This approach, which is currently implemented via open-source software packages such as SasView^14^ and SASFit,^15^ has two key limitations, namely the limited number of models for which fitting can be performed and approximations induced by inherent properties of the selected models and closure relations.^16^

On the other hand, MC simulations have become increasingly common for the analysis of SAXS curves, as this approach allows the actual equilibrium distribution of the system to be obtained for any desired interaction potentials. Machine learning (ML) algorithms have also been used in conjunction with MC simulations, which can be deployed to generate SAXS curves as training data for the ML, in order to predict various sample properties from the SAXS curves. More specifically, ML models have been utilized for the prediction of the structure and structural parameters from a given SAXS profile or the prediction of scattering data from relevant input parameters.^17^ For instance, the Computational Reverse-Engineering Analysis for Scattering Experiments (CREASE) tool uses a genetic algorithm (GA) to reconstruct three-dimensional (3D) structures for given SAS patterns and has demonstrated the ability to handle changing form and structure to provide information such as pair correlation functions of the sample.^18^ The CREASE-GA workflow was also subsequently fitted with an ML surrogate, accelerating the determination of sample information such as micelle dimensions and chain configurations of soft matter systems for an input SAS profile by over 95%.^19^ In addition, the Scattering AI Analysis (SCAN) tool is capable of directly predicting an appropriate form factor model for a given SAS profile with up to 97.3% accuracy using a combination of tree-based algorithms and neural networks.^20^ Artificial neural networks (ANNs) trained on noise-augmented SAXS profiles of nucleic acids and folded and unfolded proteins generated from experimentally determined models were also successfully employed to estimate the molecular weights and radii of gyration of these macromolecules from the corresponding SAXS profiles.^21^

While the aforementioned work has focused on the form factor, *P(q)*, of a SAXS profile, significant efforts have also been made to study and handle variations in the *S(q)* profile using ML methods. The CREASE tool was recently modified to obtain accurate reconstructed 3D structural arrangements and form factors in the presence of interparticle interactions.^18^ In addition, an ML approach based on Gaussian processes (GPs) was used to estimate colloidal interaction parameters from synthetic *S(q)* profiles and achieved good results.^22^ While the GP approach is effective in the case mentioned above and for other similar systems, GPs generally scale poorly with the dimensionality of the training data and the size of the training set,^23^ which can be overcome by using alternate ML algorithms such as ANNs.

In this work, we demonstrate a proof of concept for using an ANN trained on SAXS curves obtained from MC simulations of AuNPs to estimate colloidal interaction parameters, the effective macroion valency (*Z*_eff_), and the Debye length (κ^–1^), as described by the DLVO theory. Optimization was also carried out to determine the range of *q* values that would provide the best predictive performance for *Z*_eff_ and κ^–1^. Subsequently, Markov chain Monte Carlo (MCMC) sampling augmented with an ANN surrogate model was used to sample a defined parameter space in order to find the maximum a posteriori (MAP) estimates for *Z*_eff_ and κ^–1^ corresponding to an experimentally obtained SAXS profile of AuNPs and provide principled estimates of the confidence in the fitting and information about any possible correlation between parameters.

## Materials and Methods

### Gold Nanoparticle (AuNP) Synthesis and Ligand Exchange Procedure

The AuNPs used in this study were synthesized using the protocol reported by Yang et al.^11^ Briefly, 0.5 mmol hydrogen tetrachloroaurate(III) hydrate (HAuCl_4_·3H_2_O, 95%, Sigma-Aldrich) was dissolved in 40 mL of a 1:1 volume mixture of oleylamine (C18 content: 80 – 90%, Acros Organics) and *n*-octane (97%) in a 100 mL jacketed round-bottom flask. The mixture was sonicated in an inert argon atmosphere for 10 min to ensure complete dissolution of the HAuCl_4_. The flask was then connected to a temperature-controlled circulating bath (Grant Instruments, GR150-R2), and the contents of the flask were allowed to equilibrate to 20 °C. The reducing solution was prepared by dissolving 0.5 mmol *t*-butylamine-borane complex (97%) in 1 mL of oleylamine and 1 mL of *n*-octane before it was rapidly injected into the precursor solution under vigorous stirring. The reaction was left to run for 2 h at 20 °C in an argon atmosphere before the reaction was quenched via the addition of 30 mL of acetone. The AuNPs were washed with ethanol, collected via centrifugation at 10,000 rpm for 10 min, and redispersed in dichloromethane. The washing protocol was repeated 3 times before the obtained AuNPs were dried overnight in a vacuum desiccator prior to the ligand exchange procedure. 0.2 mmol 11-mercapto-1-undecanesulfonate (MUS) was dissolved in 10 mL of dichloromethane by vigorous mixing at room temperature for 10 min, after which 30 mg of the oleylamine-capped AuNPs was dissolved in 5 mL of dichloromethane, injected into the thiol mixture, and allowed to react for 6 h at room temperature. The ligand exchange reaction was terminated by first evaporating the solvent mixture with a rotary evaporator. The AuNPs were subsequently redissolved in approximately 10 mL of ultrapure water and purified using an Amicon Ultra-15 centrifugal filter unit (Merck Millipore, 10 kDa MWCO). These AuNPs were redispersed and passed through the filter unit 3 times to remove any excess free ligands in solution before being dried overnight in a vacuum desiccator prior to subsequent experiments.

### AuNP Small-Angle X-ray Scattering (SAXS)

SAXS experiments were carried out on a Ganesha 300XL (SAXSLAB) at 20 °C under vacuum with a high-brilliance microfocus Cu source (λ = 1.54 Å) for exposure durations of 1 h (*q* range: 0.015–0.65 Å^–1^). The beam center and the sample-to-detector distance were calibrated using the positions of diffraction peaks from a standard silver behenate powder before the scattering experiments. Samples were loaded into borosilicate glass capillaries and sealed using hot glue before being loaded into the measurement chamber. Two-dimensional (2D) SAXS patterns were collected using a Pilatus 300 K solid-state photon-counting detector with a 2 mm beam stop. The obtained 2D SAXS patterns were then radially averaged around the direct beam position using SAXSGUI software to obtain one-dimensional (1D) SAXS patterns. SAXS patterns were obtained for two samples, namely aqueous solutions of 5 and 40 mg/mL MUS-AuNPs. The 5 mg/mL MUS-AuNP sample was sufficiently dilute and was assumed to only have contributions from the form factor (i.e., *I*(*q*) ≈ *P*(*q*)). The obtained SAXS curves were fitted using SASFit, and the best-fit diameter was found to be 3.97 nm, which was the value used in the subsequent Monte Carlo simulations.

### Monte Carlo (MC) Simulations

The MC simulations were initialized by randomly assigning coordinates to 5000 spherical particles within a defined space, and interparticle interactions were modeled using the DLVO theory. More specifically, interparticle interactions were considered to be a pairwise sum of van der Waal’s attraction and electrostatic repulsion and were calculated for sampled values of *Z*_eff_ and κ^–1^ using eqs 1, 2, and 3.^15^
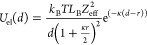
1
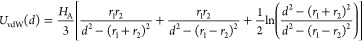
2
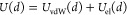
3where *U*(*d*) is the DLVO interaction potential, *U*_vdW_ is the van der Waals attraction potential, *U*_el_ is the electrostatic repulsion potential, *r* is the radius of the spheres, *d* is the center-to-center distance between the two spheres, *H*_A_ is the Hamaker constant, *k*_B_ is the Boltzmann constant, *T* is the temperature, and *L*_B_ is the Bjerrum length.

The MC simulation was then iterated by moving a randomly selected particle and calculating the total free energy within the system. The simulation temperature was set at 297K, and particle movements were accepted if it lowered the total free energy within the system. If the particle movement increased the total energy within the system, the particle movement was accepted with a probability given by the Boltzmann factor. In order to determine the equilibrium particle distribution, values of total free energy were compared every 100,000 MC steps, and the simulation was considered to have reached equilibrium if the difference in total free energy was less than 1% for 100,000 MC steps. After the MC simulation reached equilibrium, the simulation was allowed to run for another 50,000 iterations, from which 1000 sets of particle coordinates were randomly selected to calculate a radial distribution function (RDF).^24^ The structure factor, *S*(*q*), was calculated using eq 4.^25^
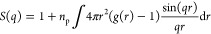
4where *S*(*q*) is the scattering structure, *n*_p_ is the particle number density, *g*(*r*) is the radial distribution function, *r* is the distance from a reference particle, and *q* is the scattering vector.

The form factor, *P*(*q*), was measured experimentally and approximated using a SAXS measurement of a dilute, 5 mg/mL aqueous solution of MUS-AuNPs, allowing simulated SAXS curves to be calculated by using eq 5
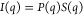
5A total of 250,000 simulated SAXS curves were obtained from the Monte Carlo simulations over a *q* range of 0.012 to 0.501 Å^–1^ by sampling combinations of *Z*_eff_ and κ^–1^ ranging from 10.0–70.0 and 3.0 nm −7.0 nm, respectively.

### Artificial Neural Network (ANN) Model Architecture and *q*_cutoff_ Optimization

The simulated SAXS curves were split into training, test, and validation sets in the ratio of 8:1:1. The training set was used to train a dense ANN, which takes a SAXS curve as input and outputs predicted values of *Z*_eff_ and κ^–1^ for the input SAXS curve. The ANN architecture consisted of 8 hidden layers, with the initial hidden layer containing 512 nodes and the number of nodes halving every subsequent hidden layer. The input layer contained a number of nodes corresponding to the number of input *q* values, which was varied to optimize the range of *q* values used for training, while the output layer contained two nodes corresponding to the two predicted quantities, *Z*_eff_ and κ^–1^. The ANN was trained with a rectified linear unit (ReLU) activation function at a learning rate of 0.001 with a loss function of mean squared error (MSE), and training was stopped when validation loss did not decrease for 10 consecutive epochs. Subsequently, the performance of the trained ANN was evaluated on a test set containing 25,000 simulated SAXS curves. All models are available at https://github.com/mdi-group/MCMC/.

### Training the ANN Surrogate Model and Inverse Markov Chain Monte Carlo (MCMC) Sampling with the Surrogate ANN model

The simulated SAXS curves were split in the same ratios as discussed above to train a dense ANN, which takes *Z*_eff_ and κ^–1^ as inputs and outputs the SAXS curve associated with the input values of the two parameters. The optimized ANN architecture consisted of 4 hidden layers, with the first layer containing 128 nodes and the rest of the hidden layers containing 512 nodes each. The output layer for the ANN contained 225 nodes, which corresponded to the predicted points on a SAXS profile for the input parameters. The ANN was trained with a ReLU activation function at a learning rate of 0.001 with a loss function of mean squared error (MSE), and training was stopped when the validation loss did not decrease for 10 consecutive epochs.

The trained ANN was then used as a surrogate for the MC simulations of the SAXS curves in a Markov chain Monte Carlo (MCMC) procedure to estimate the generating parameters of a given SAXS curve, as the ANN runs approximately 2000 times faster than the MC simulations. The MCMC sampling procedure explores sets of possible parameters (*Z*_eff_, κ^–1^) to ascertain how well each set of parameters explains the observed SAXS curve by using the ANN to generate the SAXS curve before estimating the likelihood of the parameter combination (i.e., how well the generated curve fits the data) by using an χ^2^ likelihood function shown in eq 6
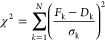
6where *F*_k_ is the predicted scattering value, *D*_k_ is the measured scattering value, and σ_k_ is the uncertainty in the measurement of the *k*th bin.

The MCMC sampling was initialized with uniform priors for *Z*_eff_ and κ^–1^ over the range of 10.0–70.0 and 3.0–7.0 nm, respectively, and outputs a maximum a posteriori (MAP) estimate as well as a probability density function, which maps the combinations of parameters against the goodness of fit, which provides insights into the confidence in the fitted parameters, reveals any correlations between parameters, and accounts for the possible presence of multiple local minimum solutions. All MCMC sampling was performed using the emcee package.^26^

## Results and Discussion

### Comparison between SAXS Curves Obtained from Monte Carlo Simulations and Analytical Methods

SASFit and SasView are existing software packages that facilitate the implementation of analytical methods to perform curve fitting for an input SAXS curve to obtain colloidal interaction parameters. More specifically, the software packages compute SAXS curves for a selected pair of form and structure factor models and performs least-squares curve fitting to obtain parameters corresponding to a SAXS curve with the smallest χ^2^ difference to the input SAXS curve. However, successful implementation of the workflow described is heavily dependent on the assumptions made in the selected models. For instance, the Hayter–Penfold RMSA is a structure factor model, which is suitable for modeling interactions between charged particles experiencing screened Coulomb repulsion forces, but this potential is only valid for colloid systems with interaction parameters within a certain operating envelope.^27^ However, the colloidal interaction parameters in systems of interest may not always align with this scope, thereby limiting the general applicability of this model.

**Figure 1:**
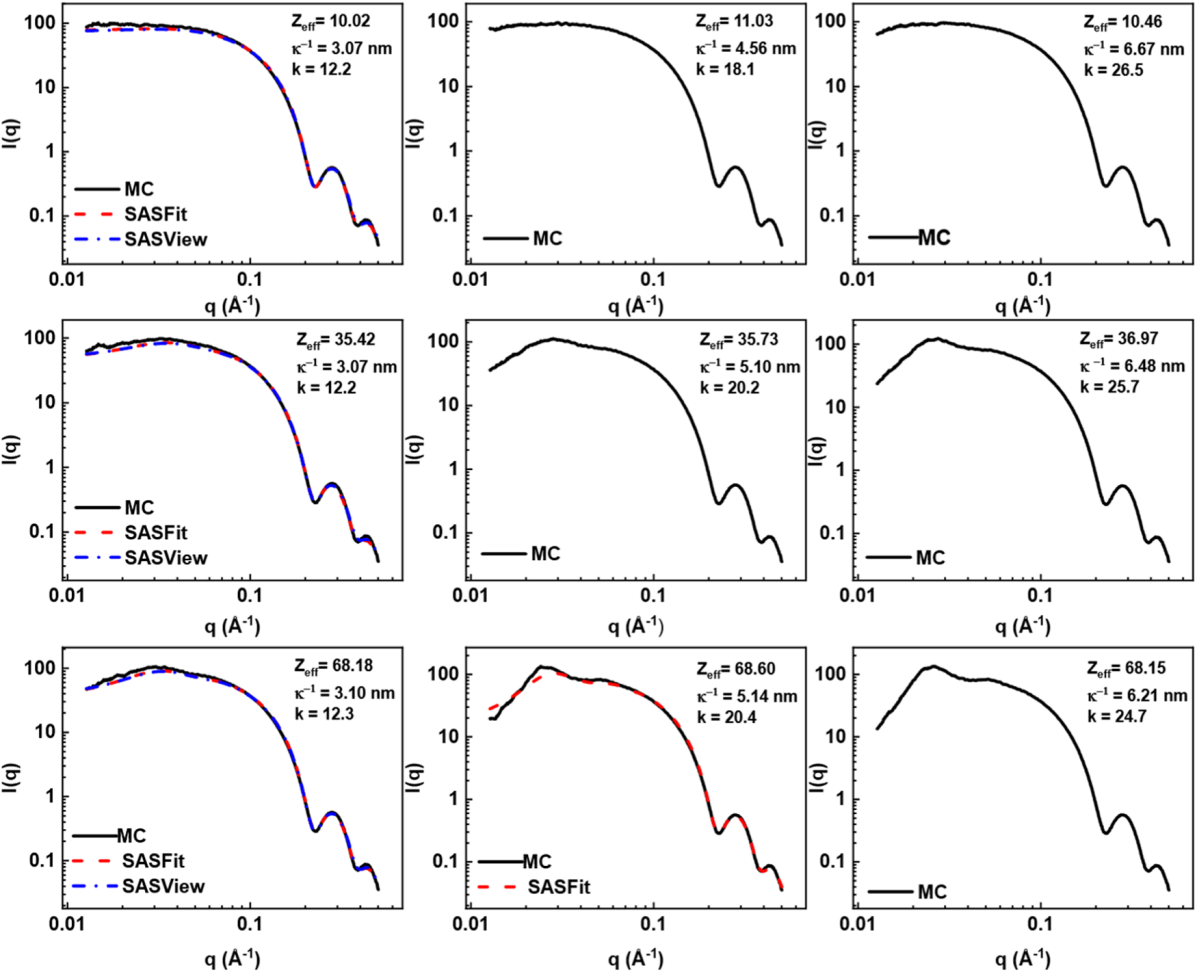
SAXS curves obtained from the Monte Carlo simulations, SASFit, and SasView over the sampled range of *Z*_eff_ and κ^–1^ values.

Figure 1 shows the SAXS curves obtained from the Monte Carlo simulations over the sampled range of *Z*_eff_ and κ^–1^ values as well as the SAXS curves obtained from the software packages using identical *Z*_eff_ and κ values. As shown in Figure 1, both analytical software packages were able to output SAXS curves for κ^–1^ ∼3 nm, which was surprising considering that the Hayter–Penfold RMSA model is established to only be valid for colloidal systems where the product of the Debye length and the particle diameter, *k*, is ≤6. Nonetheless, the SAXS curves generated from the software packages are in good agreement with the curves obtained from Monte Carlo simulations, suggesting that the Monte Carlo-based procedure for obtaining SAXS curves provides a comparable alternative to the existing analytical approach.

However, the software packages were generally unable to output SAXS curves for κ ≥ 4.5 nm, with the exception of SASFit for κ^–1^ = 5.14 nm. This result is consistent with the aforementioned operating envelope, by which the Hayter–Penfold RMSA model is limited. On the other hand, the Monte Carlo approach was able to simulate SAXS curves for κ^–1^ ≥ 4.5 nm for a range of *Z*_eff_ values. The appearance of an increasingly prominent downward curvature at the low*-q* region, which is consistent with the presence of stronger interparticle repulsion,^28^ is also observed in plots with increasing *Z*_eff_ values for similar values of κ^–1^.

### Training and Validation of an Artificial Neural Network (ANN) for the Prediction of *Z*_eff_ and κ^–1^ from SAXS Curves

**Figure 2:**
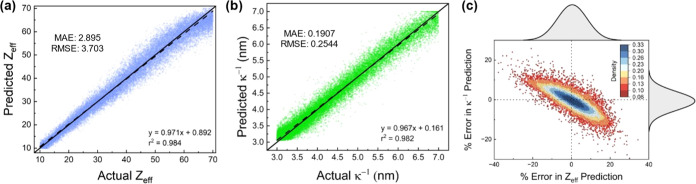
(a) Scatter plots of predicted *Z*_eff_ values against actual *Z*_eff_ values obtained from the ANN. (b) Scatter plot of predicted κ^–1^ values against actual κ^–1^ values obtained from the ANN. The solid lines are the lines of best fits corresponding to the scatter plots, and the dotted lines correspond to the diagonal *y* = *x*, which serves as a guide to the eye. (c) Scatter plots of the percentage errors associated with the predictions of *Z*_eff_ and κ^–1^ with marginal histograms showing the distribution of errors. The dashed lines correspond to *x* = 0 and *y* = 0 and serve as a guide to the eye.

The SAXS curves obtained from Monte Carlo simulations were used to train an ANN to predict *Z*_eff_ and κ^-1^ from a given SAXS curve. The performance of the ANN was evaluated on a test set containing 25,000 curves and is summarized in Figure 2. Figure 2a,b shows the scatter plots of the ANN-predicted values against the ground truth values for *Z*_eff_ and κ^–1^, respectively. The *r*^2^ and RMSE values for the fitted linear regression model for the scatter plots were found to be 0.984 and 3.703 for *Z*_eff_ and 0.982 and 0.2544 nm for κ^–1^. These values, when taken together, suggest that the ANN algorithm can offer accurate predictions of both *Z*_eff_ and κ^–1^ for unknown SAXS curves within the bounds of the training space. The prediction errors were also calculated as a percentage and are shown in Figure 2c as a scatter plot with the error distribution plotted as marginal histograms. The prediction errors follow a Gaussian distribution with a mean prediction error close to 0% for both *Z*_eff_ and κ^–1^, further highlighting the capability of the ANN algorithm to predict the values of *Z*_eff_ and κ^–1^ accurately. The distribution of prediction errors shown in Figure 2c also reveals that the ANN algorithm tends to overpredict *Z*_eff_ when underpredicting κ^–1^ and vice versa. It should be noted that *Z*_eff_ and κ^–1^ are not independent of each other, as illustrated in Figure 1, where an increase in either parameter results in stronger interparticle repulsion and thus a more pronounced downward curvature at the low*-q* region of the curve. This highlights a key limitation of extracting colloidal interaction parameters from SAXS curves, which is that a given SAXS curve is not necessarily unique for a combination of *Z*_eff_ and κ^–1^. In order to overcome this limitation, an inverse model going from parameters to curves that considers sets of possible solutions and the likelihood of these solutions rather than single solutions was developed and is discussed later in this paper. Nonetheless, the ANN algorithm is able to predict *Z*_eff_ and κ^–1^ from a given SAXS curve with prediction errors largely within ±20%.

### Optimization of the *q* Cutoff Used for ANN Training and Comparison between Values of *Z*_eff_ and κ^–1^ Obtained from Analytical Methods and ANN Prediction

**Figure 3:**
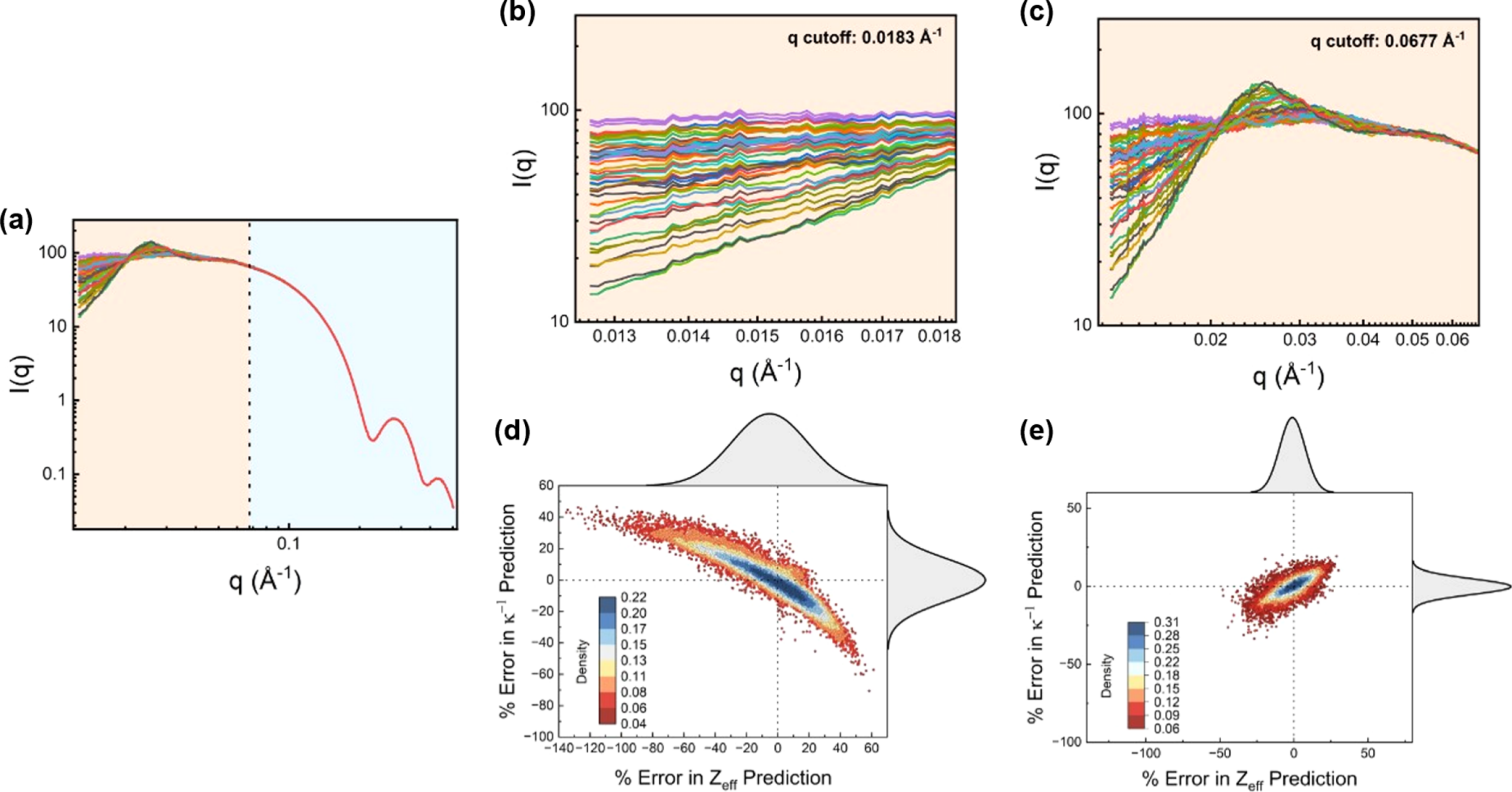
(a) Plot of 50 randomly selected SAXS curves obtained from the Monte Carlo simulations. (b, c) Plot of the same 50 randomly selected SAXS curves with *q*_cutoff_ values of 0.00183 and 0.0677 Å^–1^, respectively. (d, e) Scatter plot of the percentage errors associated with the predictions of *Z*_eff_ and κ for ANNs trained with *q*_cutoff_ values of 0.00183 and 0.0677 Å^–1^, respectively, with marginal histograms showing the distribution of errors. The dashed lines correspond to *x* = 0 and *y* = 0 and serve as a guide to the eye.

The SAXS curves were observed to overlap in the high*-q* region (*q* > 0.1 Å^–1^), while significant variation in *I*(*q*) was only observed at the low*-q* region, as illustrated in the 50 randomly selected SAXS curves obtained from the MC simulations shown in Figure 3a. This suggests that utilizing the entire range of *I*(*q*) values for each generated curve may not be necessary for training the ANN algorithm, as fitting to a very small difference at high *q* could result in significant overfitting of the model. Thus, the work done in this section was aimed at identifying the cutoff value for *q* (*q*_cutoff_), which can reduce the computation cost incurred during training while delivering the same prediction performance. Figure 3b,c shows the 50 randomly selected SAXS curves with *q*_cutoff_ values of 0.00183 and 0.0677 Å^–1^, respectively, which were used for ANN training, while Figure 3d,e shows the percentage error in the prediction of *Z*_eff_ and κ^–1^ for the ANNs trained with *q*_cutoff_ values of 0.00183 and 0.0677 Å^–1^, respectively. Details of all the trained ANNs with different *q*_cutoff_ values can be found in the Supporting Information (Figures S4–S15). It was found that the best model performance in the validation set was observed at a *q*_cutoff_ value of 0.0677 Å^–1^.

Table 1 shows the absolute errors associated with values of *Z*_eff_ and κ^–1^ obtained analytically, with the ANN models trained with *q*_cutoff_ values of 0.0677 Å^–1^ for the SAXS profiles shown in Figure 1. As discussed earlier, the analytical approach of obtaining values of *Z*_eff_ and κ^–1^ is only valid for a certain operating envelope (*k* ≤ 6), which explains the lack of predicted *Z*_eff_ and κ^–1^ values for κ^–1^ ≥ 4.5 nm in Table 1 as well as the large percentage errors in the values obtained from SASFit and SasView. The calculated percentage errors presented in Table 1 show that the ANN-predicted values have significantly smaller percentage errors as compared to those obtained via analytical methods, with the exception of the predicted value for *Z*_eff_ = 68.2, where the ANN-predicted values had an error of 7.26%, while the SasView-predicted value had a prediction error of 1.10%. However, it should be noted that the two parameters, *Z*_eff_ and κ^–1^, are obtained as a pair, and while the SasView-predicted value of *Z*_eff_ had a small percentage error, the corresponding κ^–1^ value obtained from SasView had 22.0% error, which is significantly higher than the error of 3.61% provided by the ANN.

**Table 1:** Percentage Errors Associated with the Analytical and Predicted Values of *Z*_eff_ and κ^–1^

*Z*_eff_				κ^–1^ (nm)			
Actual value	ML prediction (%)	SASFit (%)	SasView (%)	Actual value	ML prediction (%)	SASFit (%)	SasView (%)
10.0	0.183	91.9	55.7	3.07	3.46	70.3	36.7
11.0	23.5			4.56	7.25		
10.5	26.8			6.67	8.80		
35.4	10.9	39.3	43.3	3.07	3.92	5.25	45.1
35.7	15.9			5.10	6.16		
37.0	8.17			6.48	2.67		
68.2	7.26	13.2	1.10	3.10	3.61	51.7	22.0
68.6	2.59	15.1		5.14	4.76	39.9	
68.2	10.1			6.21	1.80		

### Applying an Inverse Model Using a Markov Chain Monte Carlo (MCMC) Sampling with an ANN Surrogate Model on an Experimentally Obtained SAXS Curve

As discussed earlier, combinations of different *Z*_eff_ and κ^–1^ values do not necessarily result in unique SAXS profiles, which means that multiple pairs of *Z*_eff_ and κ^–1^ values could result in identical SAXS curves. This motivates the formulation of an inverse model capable of analyzing a given experimental SAXS curve by exploring within a specified parameter domain to derive interaction parameters by obtaining curves congruent with the experimental data. One approach to solving inverse problems is the Bayesian inference, which provides a probabilistic framework for parameter estimation using Bayes’ theorem to derive the posterior distribution. However, the posterior distribution is often not obtainable analytically and can be obtained only numerically using sampling techniques such as Markov chain Monte Carlo (MCMC) methods. One key benefit of using MCMC sampling in this work is the ability to incorporate the variance associated with the SAXS measurement into the uncertainty of the estimated parameters in a weighted manner by propagating the uncertainty of the SAXS measurement.^29^

**Figure 4:**
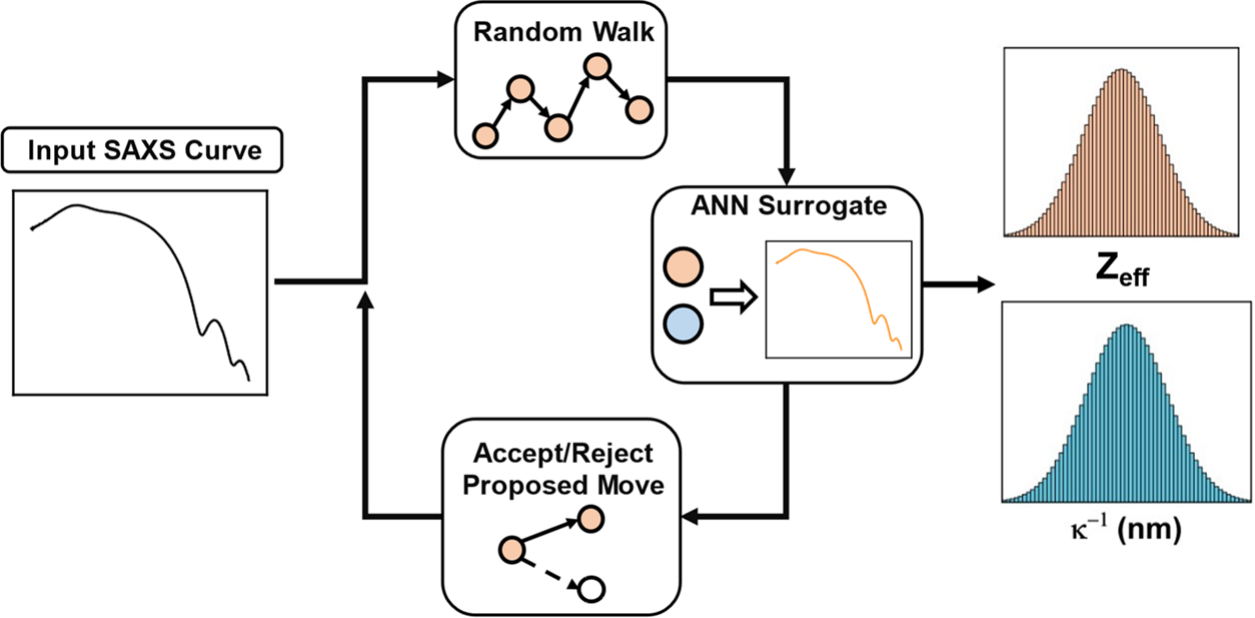
Schematic depicting the workflow for the MCMC sampling procedure, which obtains the posterior distributions for *Z*_eff_ and κ for an input SAXS curve.

Figure 4 depicts the workflow for the MCMC sampling procedure, which was used to determine maximum a posteriori (MAP) estimates for *Z*_eff_ and κ^-1^ corresponding to the input SAXS curve. The sampling procedure was initialized by defining a uniform prior for both *Z*_eff_ and κ^–1^ and a “walker”, which represents a combination of parameters within the defined parameter space. The “walker” is allowed to make random walks within the parameter space, and the random moves are accepted or rejected, depending on the log-likelihood evaluated for the “walker”. Conventionally, the SAXS profile used to compute the log-likelihood would be obtained from MC simulations, which are slow and computationally expensive due to the complex and iterative nature of the atomistic simulations involved in this work. Thus, a trained ANN was used as a surrogate model to predict a SAXS curve for a given pair of *Z*_eff_ and κ^–1^ values, effectively reducing the computational resources required to run the MCMC. After the Markov chain has converged, the posterior distribution can be visualized as a histogram by sampling the walkers. This histogram provides insights into the distribution of *Z*_eff_ and κ^–1^ values, which best explain the input SAXS curve, facilitating the identification of regions within the parameter space which correspond to solutions.

**Figure 5:**
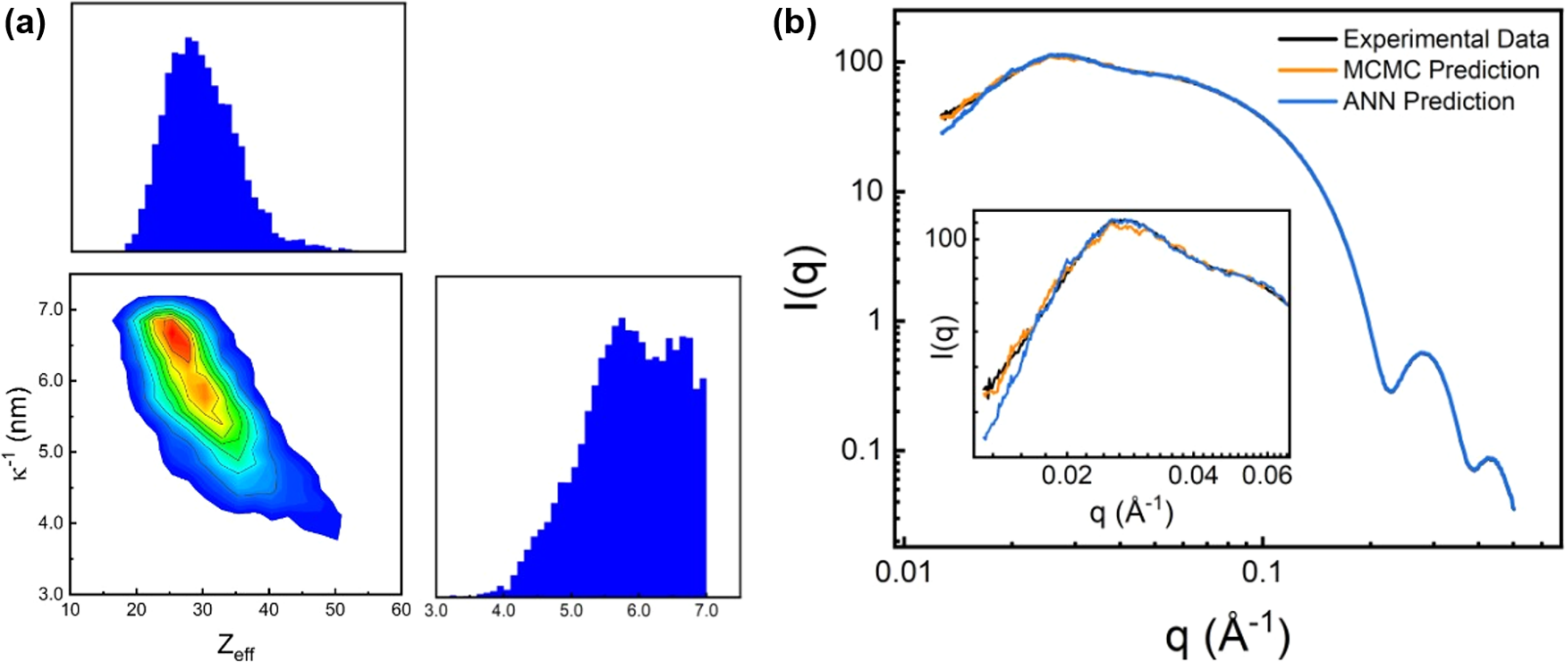
(a) Corner plot showing the posterior probability distribution sampled by the MCMC method for the experimentally obtained SAXS curve, with the diagonal panels showing the posterior probability distributions for *Z*_eff_ (top left) and κ^–1^ (bottom right). The star denotes the MAP estimation of *Z*_eff_ = 24.98 and κ^–1^ = 6.81 nm. (b) Plot of the experimentally obtained SAXS curve for MUS-AuNPs with SAXS curves obtained from the ANN and MCMC predictions.

Figure 5a shows the results obtained from an experimentally obtained SAXS curve with MCMC sampling and the ANN surrogate model. The diagonal plots in Figure 5a show the sampled posterior probability distributions obtained for *Z*_eff_ and κ^–1^, while the off-diagonal plot shows a 2D projection of the probability distributions, which maps the entire solution space and illustrates any existing correlations between the two sampled parameters. The asymmetric shape of the off-diagonal plot in Figure 5a suggests a correlation between the two parameters, which is consistent with the discussion in an earlier section. The MAP estimates of *Z*_eff_ and κ^–1^ from the MCMC sampling were found to be 24.98 and 6.81 nm, respectively. These MAP estimates were used to generate a corresponding SAXS curve using the MC simulation, which is plotted in Figure 5b, together with the experimentally obtained SAXS curve as well as a SAXS curve obtained for the values of *Z*_eff_ and κ^–1^ predicted by the forward ANN model. The curves obtained from MCMC sampling and the ANN were found to be in good agreement with the experimentally obtained SAXS curve, with the MCMC outperforming the ANN as illustrated in the inset of Figure 5b.

### Comparison between Obtaining *Z*_eff_ and κ^–1^ Values Using Markov Chain Monte Carlo (MCMC) Sampling and SasView/SASFit

Apart from being employed independently to predict colloidal interaction parameters from SAXS profiles, this sampling procedure can also be used synergistically with SasView and SASFit to improve the speed and accuracy of the analytical solutions obtained from such software. In particular, SasView and SASFit are able to perform fitting using a variety of optimization algorithms, with the most widely used algorithm being the Levenberg–Marquardt optimizer due to the fast convergence speed of the algorithm. However, the Levenberg–Marquardt algorithm easily converges to a local minimum, which may not be the global minimum.^30^ In addition, the fast convergence speed of the Levenberg–Marquardt is highly dependent on an appropriately provided initial value and the complexity of the search space.^31^ Thus, this procedure could be used to obtain a suitable initial estimate of the colloidal interaction parameters of interest before carrying out an analytical refinement of the solution, provided that the solution of interest lies within the operating envelope of the interaction potential.

While the ANN approach that we present here is highly promising, it should be noted that there are some important limitations that should be considered when employing this method. First, generating the training data for the ANN via MC simulations demands more computational resources as compared to obtaining analytical solutions from SasView and SASFit. However, these computationally expensive simulations only have to be performed once, and the obtained dataset can be used to train multiple models. Second, the quality of the ANN predictions relies inherently on the quality of the underlying training data, and thus, great care should be exercised when generating training data to ensure that it is a faithful representation of the experimental data on which the model will be applied on. Third, there is a concern that the model may still predict confidently on data outside of the parameter ranges used in the training distribution, resulting in an unreliable prediction. In contrast, the analytical fitting procedures employed by SasView and SASFit have well-defined bounds and domains of applicability. Thus, there is a need to develop robust uncertainty estimations for the ANN predictions to ensure accurate parameter predictions by the ANN. Lastly, ANN models can be difficult to interpret, further complicating the difficulty of explaining unexpected results, which could be a result of genuinely new phenomena or simply a bug in the model, motivating future work on model interpretability.^32^

## Conclusions

In this work, we demonstrated a proof of concept for using ANNs to estimate colloidal interaction parameters, *Z*_eff_ and κ^–1^, from SAXS profiles obtained from MC simulations of nanoparticles subjected to different interaction strengths. As compared to the currently used analytical approach of obtaining colloidal interaction parameters, the ANN approach is not limited by assumptions made in models or closure relations, which are necessary for obtaining analytical estimates of *Z*_eff_ and κ^–1^. In addition, the trained ANN is able to provide good estimates of *Z*_eff_ and κ^–1^ for a set of test data containing 25,000 SAXS curves, with a majority of the estimates having prediction errors within ±20%. An inverse ANN was then used as a surrogate model to perform MCMC sampling and successfully was used to interpret an experimentally obtained SAXS profile. We believe that the approach described in this study, coupled with appropriate interaction potentials, can be used to study a wide range of colloidal interaction phenomena.

## Data Availability

A repository containing the data used to train the ANN and the codes used in this work has been made available on GitHub (https://github.com/mdi-group/MCMC).

## Supporting Information Available

Distribution of sampled parameters in the dataset used to train the ANN and performance of the ANN with varying *q*_cutoff_ values (PDF)
