# Supplementary material for: Predicting Colloidal Interaction Parameters from Small-Angle X-ray Scattering Curves Using Artificial Neural Networks and Markov Chain Monte Carlo Sampling
Source: JACS Au. 2024 Sep 9;4(9):3492–500. doi: 10.1021/jacsau.4c00368 (PMC11423300; doi:10.1021/jacsau.4c00368)
Supplement: Supplementary file 1 — au4c00368_si_001.pdf [file au4c00368_si_001.pdf]

SUPPORTING INFORMATION

Predicting Colloidal Interaction Parameters from Small Angle X-Ray Scattering Curves using Artificial Neural Networks and Markov Chain Monte Carlo Sampling

Kelvin Wong[1], Runzhang Qi[2,3], Ye Yang[1,3], Zhi Luo[4], Stefan Guldin[1,◊,*], Keith T. Butler[5,*]

[1] Department of Chemical Engineering, University College London, Torrington Place, London, WC1E 7JE, United Kingdom.

[2] Yusuf Hamied Department of Chemistry, Centre for Misfolding Diseases, University of Cambridge, Lensfield Road, Cambridge, CB2 1EW, United Kingdom.

[3] Langmu Bio, Building 2, 112 Jinjiadulu, Yuhang, Hangzhou 311112, China.

[4] Guangdong Provincial Key Laboratory of Advanced Biomaterials, Department of Biomedical Engineering, Southern University of Science and Technology, Shenzhen, 518055, China.

[5] Department of Chemistry, University College London, Kathleen Lonsdale Building, Gower Place, WC1E 6BS, United Kingdom.

◊ now at: Technical University of Munich, Department of Life Science Engineering Gregor-Mendel-Straße 4, 85354 Freising, Germany.

*Corresponding Authors:

Stefan Guldin: Email: s.guldin@ucl.ac.uk; guldin@tum.de

Keith T. Butler: Email: k.t.butler@ucl.ac.uk

Table of Contents

Figure S1 3
Figure S2 3
Figure S3 3
Figure S4 4
Figure S5 4
Figure S6 5
Figure S7 5
Figure S8 6
Figure S9 6
Figure S10 7
Figure S11 7
Figure S12 8
Figure S13 8
Figure S14 9
Figure S15 9
Figure S16 10

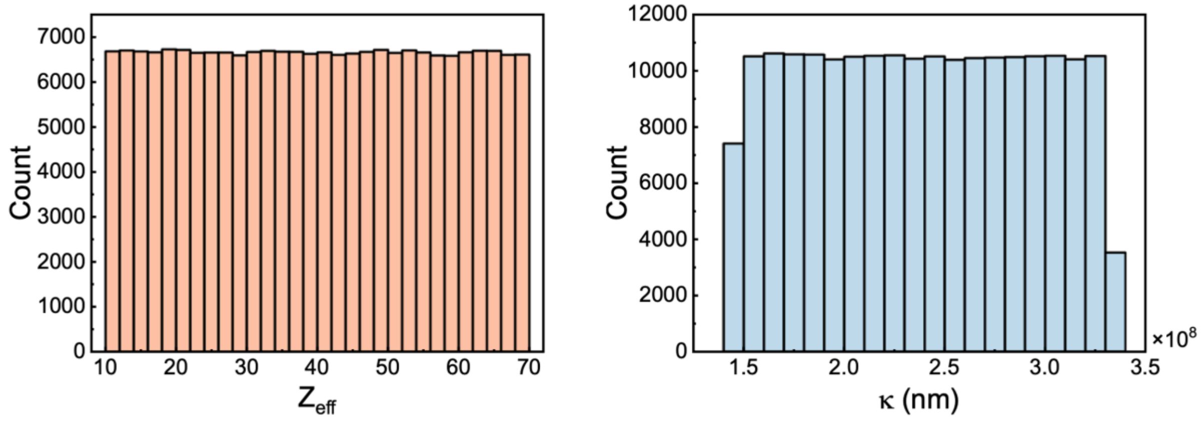

Figure S1: Distribution of sampled Z_{eff} and κ^{-1} values used in training the artificial neural network surrogate model.

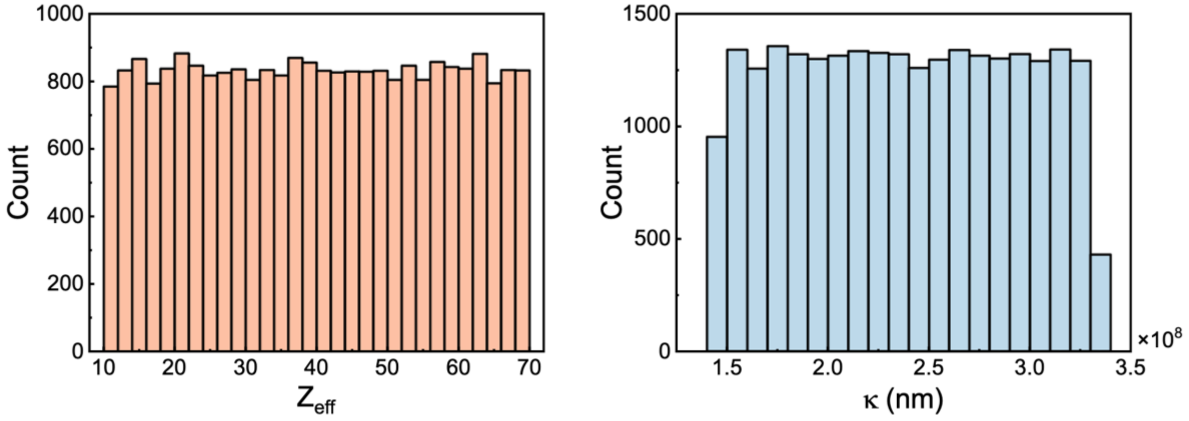

Figure S2: Distribution of sampled Z_{eff} and κ^{-1} values used in validating the artificial neural network surrogate model performance.

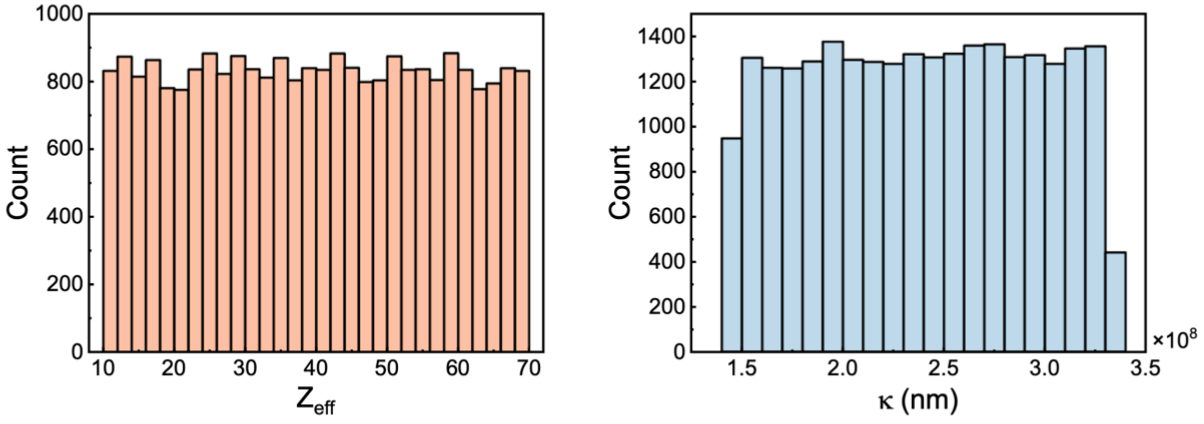

Figure S3: Distribution of sampled Z_{eff} and κ^{-1} values used in testing the artificial neural network surrogate model performance.

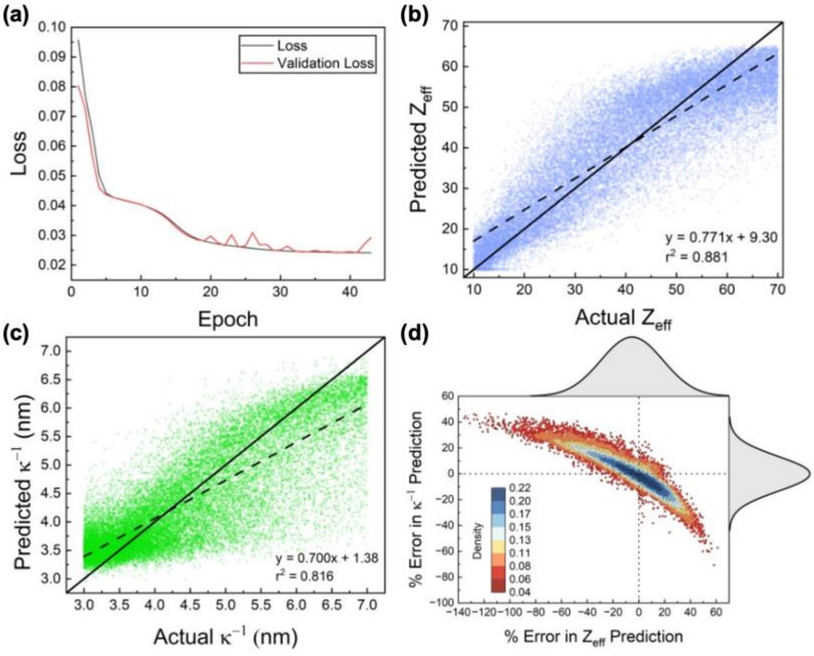

Figure S4: (a) Plot of loss and validation loss across epochs during training for ML model with q_{cutoff} of 0.0183 Å^{-1}. (b) Scatter plot of predicted Z_{eff} values against actual Z_{eff} values for a test set containing 25,000 simulated SAXS curves. (c) Scatter plot of predicted $\boldsymbol{\kappa}^{-1}$ values against actual $\boldsymbol{\kappa}^{-1}$ values for a test set containing 25,000 simulated SAXS curves. (d) Scatter plot of the percentage error in predictions of Z_{eff} and $\boldsymbol{\kappa}^{-1}$ with marginal histograms showing the error distribution.

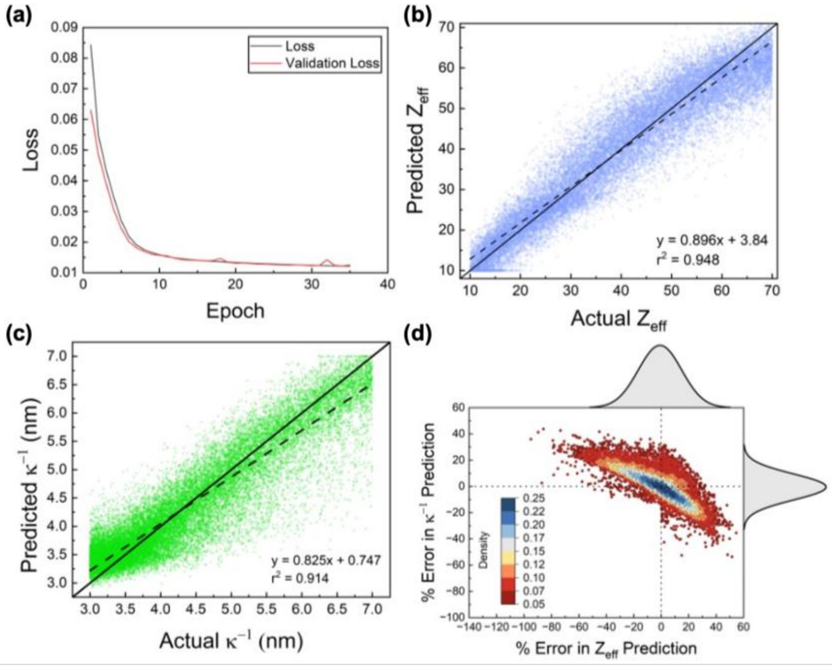

Figure S5: (a) Plot of loss and validation loss across epochs during training for ML model with q_{cutoff} of 0.0221 Å^{-1}. (b) Scatter plot of predicted Z_{eff} values against actual Z_{eff} values for a test set containing 25,000 simulated SAXS curves. (c) Scatter plot of predicted $\boldsymbol{\kappa}^{-1}$ values against actual $\boldsymbol{\kappa}^{-1}$ values for a test set containing 25,000 simulated SAXS curves. (d) Scatter plot of the percentage error in predictions of Z_{eff} and $\boldsymbol{\kappa}^{-1}$ with marginal histograms showing the error distribution.

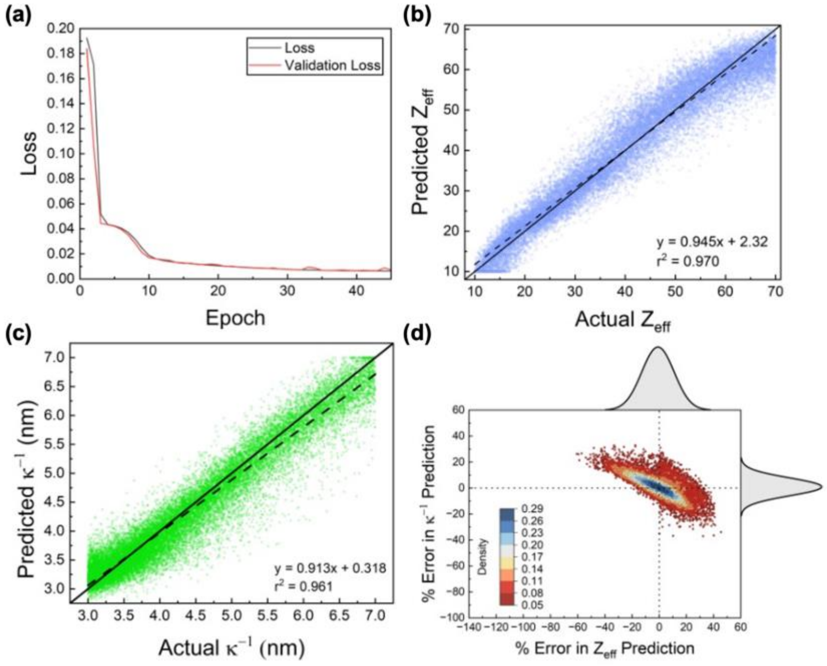

Figure S6: (a) Plot of loss and validation loss across epochs during training for ML model with q_{cutoff} of 0.0266 Å^{-1}. (b) Scatter plot of predicted Z_{eff} values against actual Z_{eff} values for a test set containing 25,000 simulated SAXS curves. (c) Scatter plot of predicted $\boldsymbol{\kappa}^{-1}$ values against actual $\boldsymbol{\kappa}^{-1}$ values for a test set containing 25,000 simulated SAXS curves. (d) Scatter plot of the percentage error in predictions of Z_{eff} and $\boldsymbol{\kappa}^{-1}$ with marginal histograms showing the error distribution.

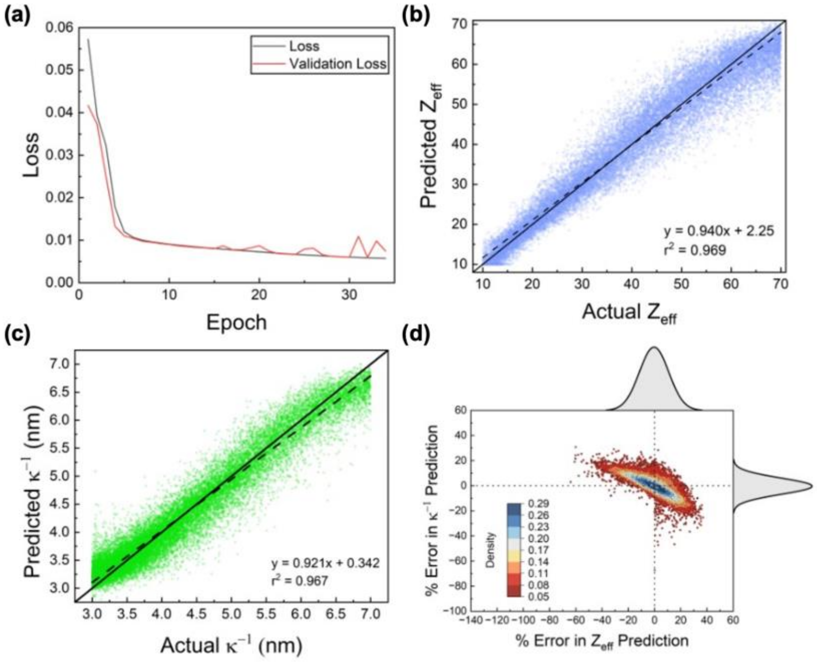

Figure S7: (a) Plot of loss and validation loss across epochs during training for ML model with q_{cutoff} of 0.0321 Å^{-1}. (b) Scatter plot of predicted Z_{eff} values against actual Z_{eff} values for a test set containing 25,000 simulated SAXS curves. (c) Scatter plot of predicted $\boldsymbol{\kappa}^{-1}$ values against actual $\boldsymbol{\kappa}^{-1}$ values for a test set containing 25,000 simulated SAXS curves. (d) Scatter plot of the percentage error in predictions of Z_{eff} and $\boldsymbol{\kappa}^{-1}$ with marginal histograms showing the error distribution.

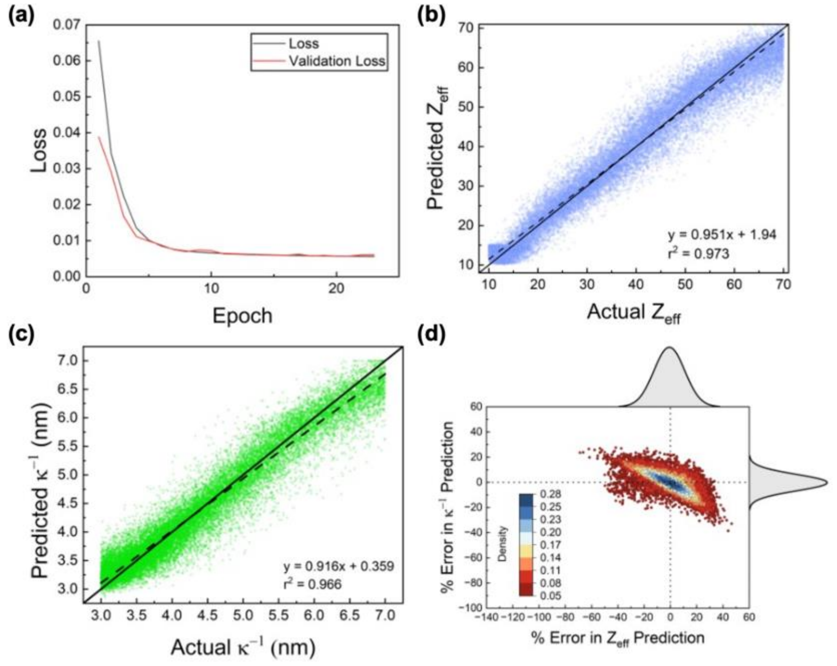

Figure S8: (a) Plot of loss and validation loss across epochs during training for ML model with q_{cutoff} of 0.0387 Å^{-1}. (b) Scatter plot of predicted Z_{eff} values against actual Z_{eff} values for a test set containing 25,000 simulated SAXS curves. (c) Scatter plot of predicted $\boldsymbol{\kappa}^{-1}$ values against actual $\boldsymbol{\kappa}^{-1}$ values for a test set containing 25,000 simulated SAXS curves. (d) Scatter plot of the percentage error in predictions of Z_{eff} and $\boldsymbol{\kappa}^{-1}$ with marginal histograms showing the error distribution.

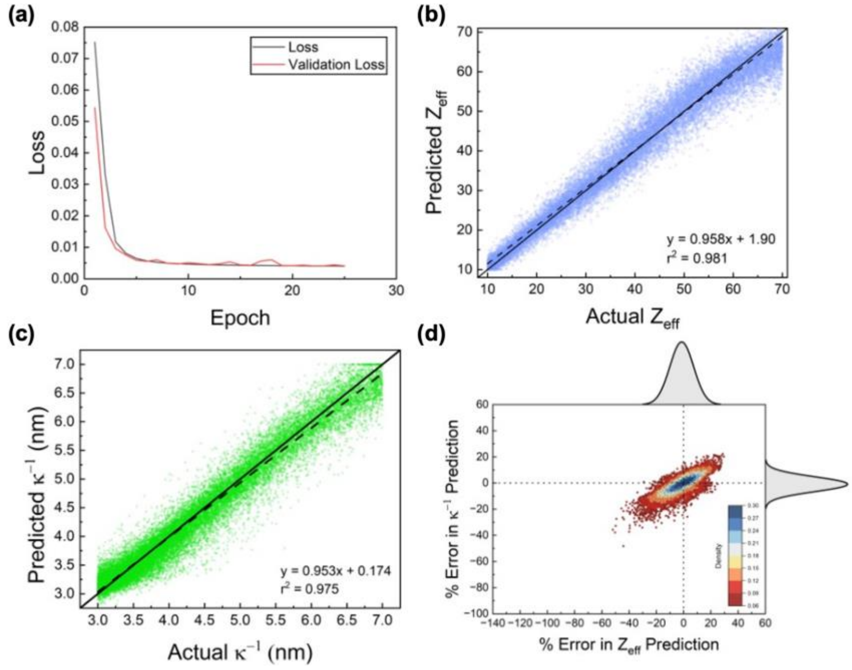

Figure S9: (a) Plot of loss and validation loss across epochs during training for ML model with q_{cutoff} of 0.0466 Å^{-1}. (b) Scatter plot of predicted Z_{eff} values against actual Z_{eff} values for a test set containing 25,000 simulated SAXS curves. (c) Scatter plot of predicted $\boldsymbol{\kappa}^{-1}$ values against actual $\boldsymbol{\kappa}^{-1}$ values for a test set containing 25,000 simulated SAXS curves. (d) Scatter plot of the percentage error in predictions of Z_{eff} and $\boldsymbol{\kappa}^{-1}$ with marginal histograms showing the error distribution.

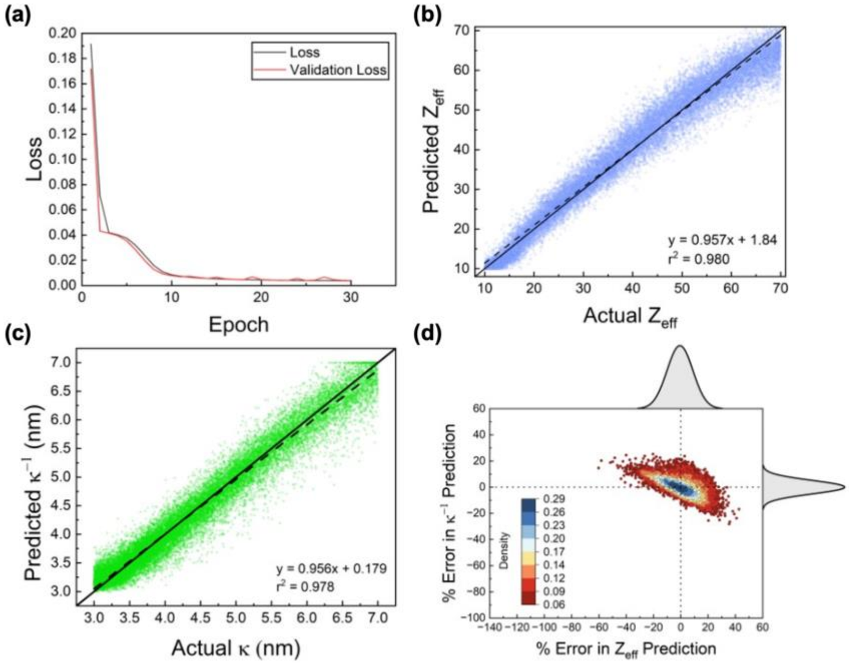

Figure S10: (a) Plot of loss and validation loss across epochs during training for ML model with q_{cutoff} of 0.0562 Å^{-1}. (b) Scatter plot of predicted Z_{eff} values against actual Z_{eff} values for a test set containing 25,000 simulated SAXS curves. (c) Scatter plot of predicted $\boldsymbol{\kappa}^{-1}$ values against actual $\boldsymbol{\kappa}^{-1}$ values for a test set containing 25,000 simulated SAXS curves. (d) Scatter plot of the percentage error in predictions of Z_{eff} and $\boldsymbol{\kappa}^{-1}$ with marginal histograms showing the error distribution.

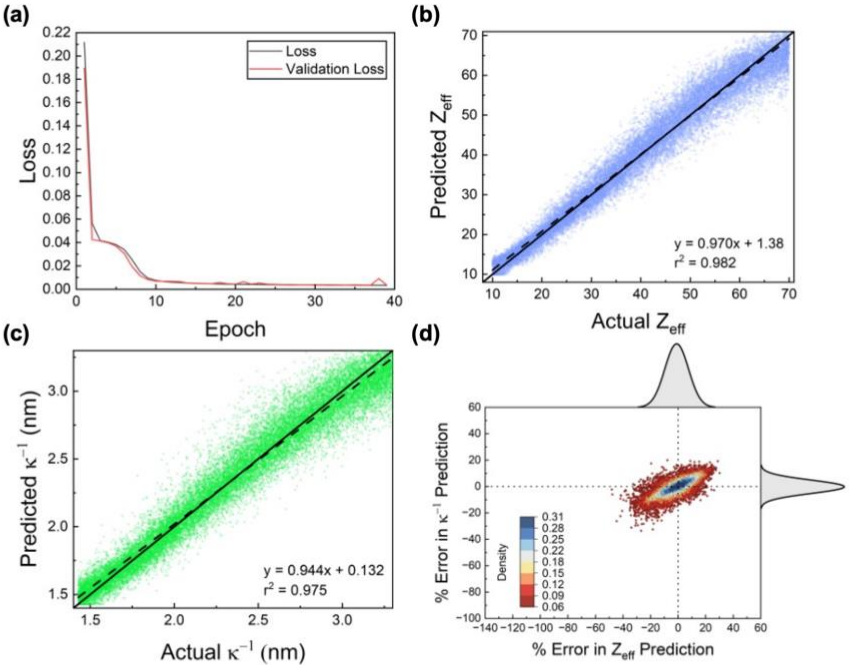

Figure S11: (a) Plot of loss and validation loss across epochs during training for ML model with q_{cutoff} of 0.0677 Å^{-1}. (b) Scatter plot of predicted Z_{eff} values against actual Z_{eff} values for a test set containing 25,000 simulated SAXS curves. (c) Scatter plot of predicted $\boldsymbol{\kappa}^{-1}$ values against actual $\boldsymbol{\kappa}^{-1}$ values for a test set containing 25,000 simulated SAXS curves. (d) Scatter plot of the percentage error in predictions of Z_{eff} and $\boldsymbol{\kappa}^{-1}$ with marginal histograms showing the error distribution.

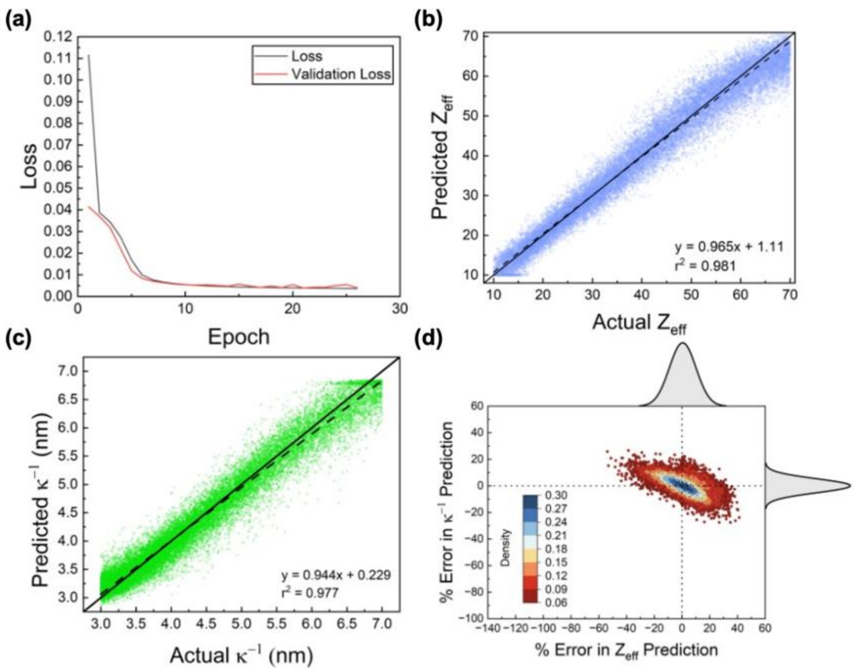

Figure S12: (a) Plot of loss and validation loss across epochs during training for ML model with q_{cutoff} of 0.0816 Å^{-1}. (b) Scatter plot of predicted Z_{eff} values against actual Z_{eff} values for a test set containing 25,000 simulated SAXS curves. (c) Scatter plot of predicted $\boldsymbol{\kappa}^{-1}$ values against actual $\boldsymbol{\kappa}^{-1}$ values for a test set containing 25,000 simulated SAXS curves. (d) Scatter plot of the percentage error in predictions of Z_{eff} and $\boldsymbol{\kappa}^{-1}$ with marginal histograms showing the error distribution.

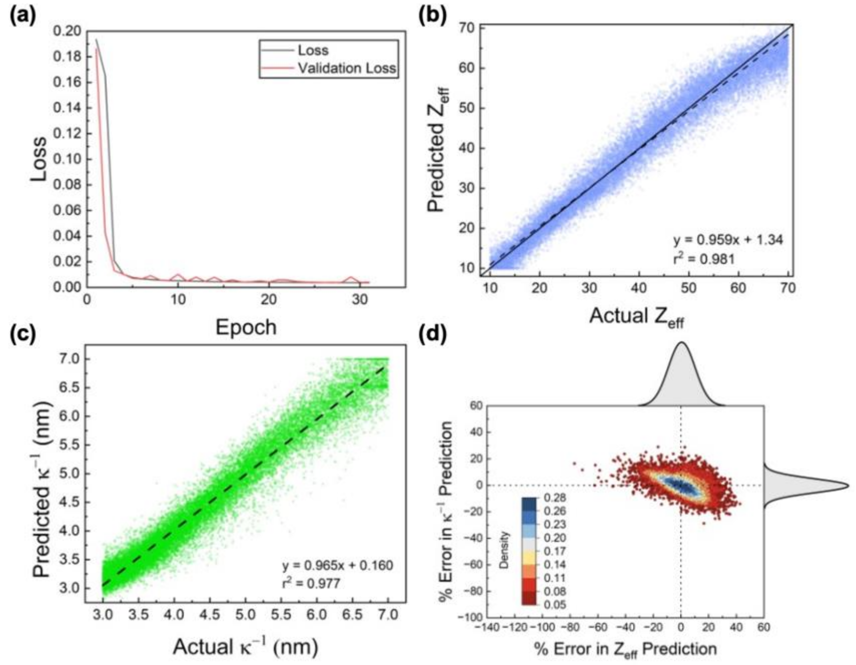

Figure S13: (a) Plot of loss and validation loss across epochs during training for ML model with q_{cutoff} of 0.0984 Å^{-1}. (b) Scatter plot of predicted Z_{eff} values against actual Z_{eff} values for a test set containing 25,000 simulated SAXS curves. (c) Scatter plot of predicted $\boldsymbol{\kappa}^{-1}$ values against actual $\boldsymbol{\kappa}^{-1}$ values for a test set containing 25,000 simulated SAXS curves. (d) Scatter plot of the percentage error in predictions of Z_{eff} and $\boldsymbol{\kappa}^{-1}$ with marginal histograms showing the error distribution.

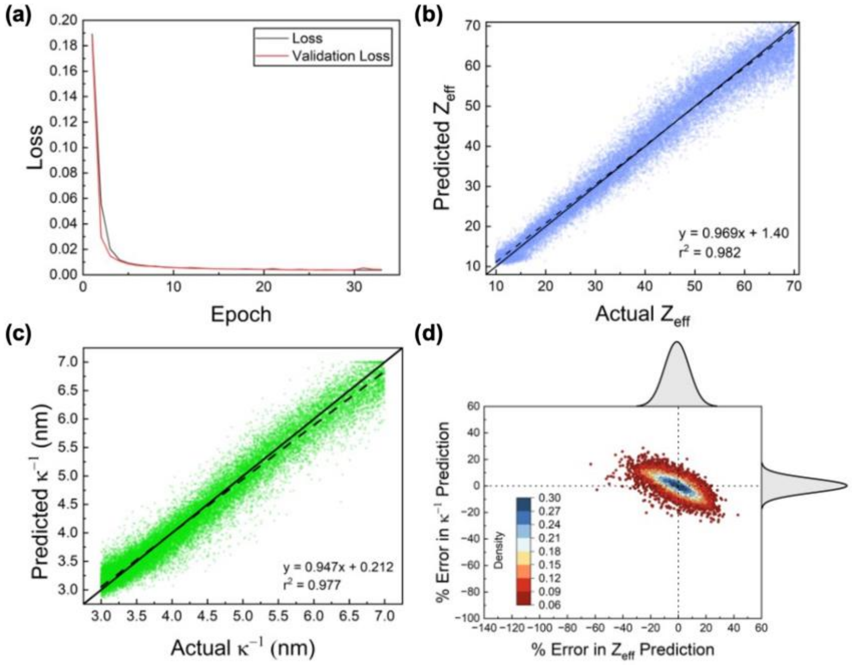

Figure S14: (a) Plot of loss and validation loss across epochs during training for ML model with q_{cutoff} of 0.119 $Å^{-1}$. (b) Scatter plot of predicted Z_{eff} values against actual Z_{eff} values for a test set containing 25,000 simulated SAXS curves. (c) Scatter plot of predicted κ^{-1} values against actual κ^{-1} values for a test set containing 25,000 simulated SAXS curves. (d) Scatter plot of the percentage error in predictions of Z_{eff} and κ^{-1} with marginal histograms showing the error distribution.

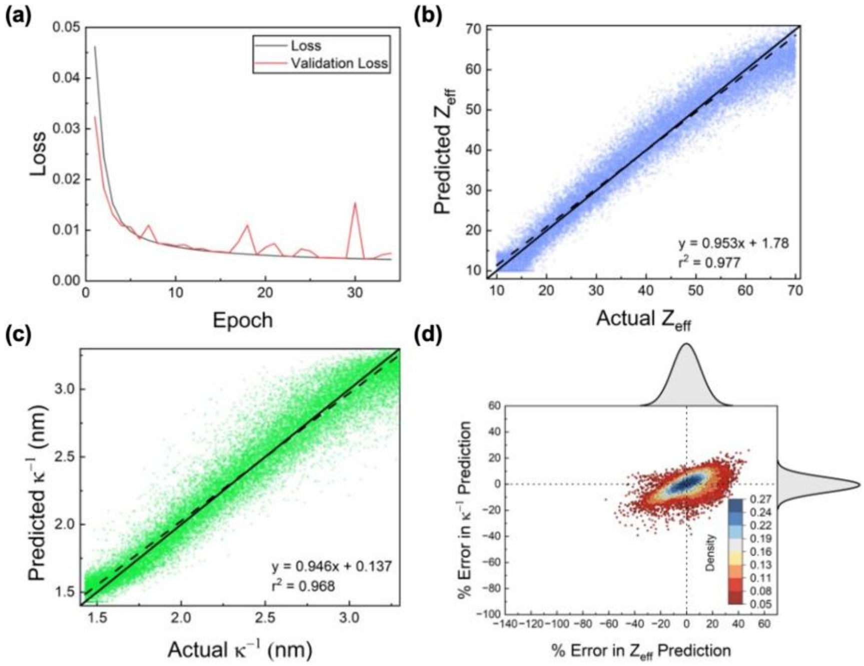

Figure S15: (a) Plot of loss and validation loss across epochs during training for ML model with q_{cutoff} of 0.502 $Å^{-1}$. (b) Scatter plot of predicted Z_{eff} values against actual Z_{eff} values for a test set containing 25,000 simulated SAXS curves. (c) Scatter plot of predicted κ^{-1} values against actual κ^{-1} values for a test set containing 25,000 simulated SAXS curves. (d) Scatter plot of the percentage error in predictions of Z_{eff} and κ^{-1} with marginal histograms showing the error distribution.

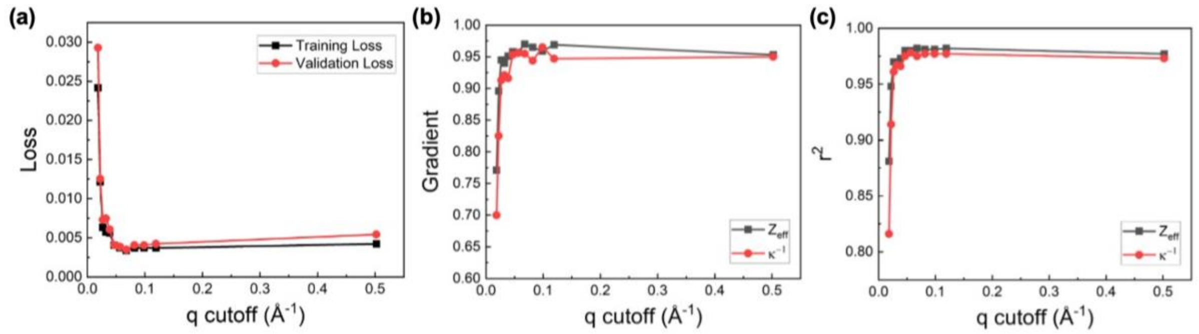

Figure S16: (a) Plot of training and validation loss for final model against q_{cutoff}. (b) Plot of gradient of the linear best fit for predicted values of Z_{eff} and $\boldsymbol{\kappa^{-1}}$ against q_{cutoff}. (c) Plot of r^2 value for the linear best fit for predicted values of Z_{eff} and $\boldsymbol{\kappa^{-1}}$ against q_{cutoff}.
